# Low appendicular skeletal muscle mass index is associated with the anthropometric variables of post-menopausal women

**DOI:** 10.1186/s12877-022-03313-y

**Published:** 2022-08-03

**Authors:** Geise Ferreira da Cruz, Tatiana Mion Lunz, Tatielle Rocha de Jesus, Mariana Braga Costa, Camila Vilarinho Vidigal, Ben-Hur Albergaria, José Luiz Marques-Rocha, Valdete Regina Guandalini

**Affiliations:** 1 Department of Integrated Health Education, Federal University of Espirito Santo, Vitoria, Marechal Campos, avenue, 1468 – Maruípe, Vitória, Espírito Santo 29040-090 Brazil; 2 Department of Integrated Education, Federal University of Espirito Santo, Marechal Campos, avenue, 1468 – Maruípe, Vitória, Espírito Santo 29040-090 Brazil; 3 Department of Social Medicine, Federal University of Espirito Santo, Marechal Campos, avenue, 1468 – Maruípe, Vitória, Espírito Santo 29040-090 Brazil

**Keywords:** Menopause, Body composition, Densitometry, Sarcopenia, Skeletal muscle

## Abstract

**Background:**

Skeletal muscle mass is a central component of body composition and its decline is enhanced during aging. We verified the association between the appendicular skeletal muscle mass index (ASMI) with the anthropometric variables, biochemical variables, and lifestyle of postmenopausal women.

**Methods:**

Cross-sectional observational study conducted with postmenopausal women. Sociodemographic, clinical, lifestyle, physical activity level, biochemical, and anthropometric markers were collected. Body composition was assessed by dual-energy densitometry. Multivariate logistic regression analysis was applied.

**Results:**

One hundred fourteen women aged in average 66.0 ± 5.8 years were evaluated. There was a significant association between ASMI and age (*p* = 0.004), body mass (*p* < 0.001), body mass index (BMI) (p < 0.001), adductor pollicis muscle thickness (APMT) (*p* < 0.001), plasma calcium levels (*p* = 0.003), calf circumference (CC), and waist circumference (WC) (p < 0.001 for both). Adjusted regression analyses revealed the influence of BMI, CC**,** and APMT in the 1st tertile of ASMI (*p* < 0.05), BMI and CC in the 2rd tertile of ASMI.

**Conclusions:**

ASMI was associated with BMI and muscle mass reserve indicators such as CC and DAPMT. In clinical practice, this indicates that simple, low-cost measures with good applicability can be used to predict and track the risk of depletion of skeletal muscle mass and consequent sarcopenia.

## Background

Skeletal muscle mass (SMM) is a central component of body composition and its decline is potentiated during the aging process, which can lead to an increase in the risk of fractures, osteoporosis, poor quality of life, functional disability, and mortality [1–3].

The main clinical approach to assess muscle depletion is the use of the appendicular skeletal muscle mass index (ASMI), which consists of the sum of the skeletal muscle mass of the arms and legs corrected for height squared [4, 5]. ASMI can be determined using indirect instruments such as magnetic resonance imaging (MRI), computed tomography (CT), dual energy x-ray absorptiometry (DXA), and bioelectrical impedance (BIA) [6]. However, due to the unavailability and cost of these instruments, anthropometric measurements have been put forward as alternatives in places or situations in which these methods are not available [7–9].

Previous studies point to the use of calf circumference (CC) as a sensitive anthropometric indicator, having been widely used for screening of low muscle mass and sarcopenia [7, 10, 11]. Likewise, body mass index (BMI) and adductor pollicis muscle thickness (APTM) have also been shown to be associated with reduced appendicular skeletal muscle mass (ASM) [12–14]. In common, these variables present the fact that they are low-cost, non-invasive, and easily reproducible in clinical practice [9, 14, 15]. In addition, factors such as age [16], gender [17], nutrition [16], physical activity [18], and lifestyle [19] have been shown to be directly related to the decline in muscle mass, especially among postmenopausal women, as a consequence of the sharp drop in estrogen at this stage [1, 20].

Despite the risks and susceptibility of comorbidities associated with reduced ASMI in the postmenopausal period [5, 12], few Brazilian studies investigated anthropometric and biochemical variables, in addition to muscle mass reserve indicators through DXA in this population. Studies on this subject are usually conducted with elderly people of both sexes, without considering menopausal status and time of menopause [21].

In this context, early identification and prevention of ASM depletion through alternative screening tools can promote better quality of life, independence, and preserved functional capacity in postmenopausal women [2, 22], since they are directly affected in the presence of sarcopenia [12]. Thus, this study investigated the association between ASMI and the anthropometic variables, biochemical variables, and lifestyle of Brazilian postmenopausal women.

## Methods

Cross-sectional, observational study with a probabilistic sample conducted with women recruited at the General Outpatient Clinic of Gynecology and Obstetrics of a University Hospital located in Vitória, Espírito Santo, Brazil, from June 2019 to March 2020.

### Population

The study included women aged between 50 and 85 years, in menopause for at least 12 months, cared for at a secondary public service. Those under Hormone Replacement Therapy (HRT), with cardiac or metallic implants that prevented the exams from being performed, and who did not respond to four attempts at telephone contact were excluded.

### Sampling and sample selection

The sample size was defined based on the number of consultations that took place at that clinic in 2018, totaling 527 consultations. After excluding duplicate consultations and women aged under 50 and over 85 years (*n =* 185), 342 women remained eligible for the study. For sample calculation, we used the software Open Source Epidemiologic Statistics for Public Health (OpenEpi Version 3.01) [23]. A confidence interval of 95% was considered with a margin of error of 5% and a prevalence of osteoporosis of 21.3% [24]. The present study is a continuation of the article “Influence of the appendicular skeletal muscle mass index on the bone mineral density of postmenopausal women” [25], in which it was necessary to apply the prevalence of osteoporosis to calculate the final sample, as validated by Luiz and Magnanini [26], resulting in a sample size of 147 women.

Sample selection was carried out through a simple random drawing of the names of the women in question, which were allocated in a list, using the software Excel® (Office 2016). The women selected were contacted by telephone and invited to participate in the study, during which the research objectives were clarified. Those who refused to participate or did not meet the inclusion criteria were replaced in a new drawing.

### Study variables and instruments

Interviews and data collection were carried out in the morning at the ELSA Brazil Research Center, in Vitória, Espírito Santo, by professionals duly trained and qualified for this purpose.

### Outcome

The mail outcome this research was ASMI, obtained from the ratio between appendicular skeletal muscle mass ASMM and height squared [5]. The ASMM were measured through Dual Energy X-ray Absorptiometry – DXA (GE Lunar Prodigy Advance®), duly calibrated, using the GE Encore software, version 14.10, configured to use the reference database of the National Health and Nutrition Examination Survey [27]. All body densitometry exams were performed by a trained radiology technician and the result interpreted and signed by a single specialist physician to avoid interobserver variation. Values were classified according to the tertiles themselves.

### Exposure variables

The anthropometric, body composition, and functional capacity variables collected were height (cm), body mass (kg), body mass index (BMI), calf circumference (CC), waist circumference (WC), dominant adductor pollicis muscle thickness (DAPMT), body fat (%), dominant handgrip strength (DHGS) and Timed Up and Go test (TUG).

Height (m) and body mass (kg) were measured according to techniques proposed by Lohman et al. [28]. BMI (kg/m2) was obtained from the ratio between body mass (kg) and height (m) squared. CC was measured at the largest calf bulge in the sitting position and the knee flexed at 90° [28]. WC was measured at the midpoint between the iliac crest and the last costal arch [29]. To measure APMT, we used the technique proposed by Lameu et al. [30], which was performed in triplicate in the dominant hand with the mean value of the three measurements considered for analysis. Body fat was measured through Dual Energy X-ray Absorptiometry – DXA.

HGS was evaluated in triplicate in the dominant hand under verbal encouragement for 5 seconds and 1-minute intervals between measurements, according to the method recommended by the “American Society of Hand Therapists” (ASHT) [31]. The maximum value was considered for analysis. The TUG test was conducted according to the technique proposed by Podsiadlo and Richardson [32], being carried out in triplicate and adopting the mean time for analysis.

Biochemical variables were obtained through venipuncture performed in the morning at the clinical analysis laboratory of the University Hospital, following a 12-hour fasting.

### Covariables

The following information was collected: sociodemographic – age (years), marital status, education (years), and self-reported race/color (white, black, brown, yellow, and indigenous) [33]; lifestyle – smoking habit, alcohol consumption, and level of physical activity; and clinical information – age at menarche, age at menopause, comorbidities, and use of medications. Adult women were those aged less than 60 years and elderly those aged over 60 years [34].

The level of physical activity was estimated using the International Physical Activity Questionnaire (IPAQ), long version [35], considering only the sum of the transport and leisure sections [36]. Women who reported performing 150 minutes or more of weekly physical activity of moderate intensity or above 75 minutes of vigorous physical activity per week were classified as active according to the guidelines on physical activity and sedentary behavior proposed by the World Health Organization [37]. Comorbidities, polypharmacy and calcium supplementation use were collected from medical records and categorized into: 1 to 3 comorbidities and 4 or more comorbidities, and polypharmacy into: 1 to 4 medications and 5 or more medications.

### Ethical aspects

The participation of individuals in this study was voluntary and was conducted in accordance with the Resolution 466 of December 08, 2012, of the National Health Council of Brazil [38]. The written informed consent was obtained from all participants. Ethical approval for this study was obtained from the Research Ethics Committee of the Federal University of Espírito Santo – Protocol n° 2.621.794.

### Statistical analysis

Descriptive analysis was expressed as means and standard deviations to describe continuous variables and as a percentage for categorical variables. The normality of the variables was analyzed using the Kolmogorov-Smirnov test. ASMI was classified according to the tertiles themselves [39]. Chi-square tests and Fisher’s Exact test were applied to verify the diference between proportions according to the ASMI tertiles. One-way Anova and Kruskal Wallis tests were applied to verify the difference between means according to the tertiles of ASMI. Multivariate logistic regression models were adjusted considering two main outcomes: 1st tertile (5.68 ± 0.31 kg/m^2^) and 2nd tertile (6.72 ± 0.28 kg/m^2^) of ASMI, taking as reference the 3rd tertile (7.78 ± 0.53 kg/m^2^). Collinear or strongly correlated variables were not included in the models. For the final model, those with *p*-values < 0.05 in the bivariate analyses were maintained. Odds ratios and their respective confidence intervals were calculated. Data were analyzed using SPSS 22.0 software and the significance level adopted for all tests was 5.0%.

## Results

**Fig. 1:**
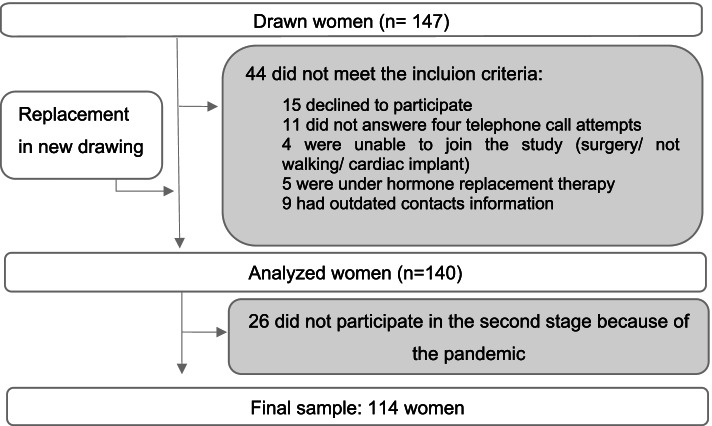
Selection flowchart

Of the 147 women drawn, 44 did not meet the inclusion criteria and were replaced in a new drawing. Although 140 women were included, the collection was interrupted because of the advance of the pandemic by the new coronavirus (SARS-CoV2), which made it impossible for 26 participants to complete the exams and resulted in 114 women in the final sample (Fig. 1).

The mean age of the women analyzed was 66.0 ± 5.8 years, with a predominance of the age group from 60 to 69.9 years (62.3%). Of these, 49.1% declared themselves brown, 41.2% had less than 4 years of schooling, and most of them had never smoked (69.3%) or consumed alcohol (63.2%). In the final sample, there was a greater proportion of sufficiently active women (53.5%), with ≤19 years of menopause (51.8%), who had one to three comorbidities (74.6%), using one to four medications (57.9%), and taking calcium supplements (59.6%) (Table 1).

**Table 1:** Distribution of sociodemographic, lifestyle, and clinical variables according to the appendicular skeletal muscle mass index of postmenopausal women

Variables *n =* 114	ASMI (kg/m^**2**^)				
	Total n (%)	1st Tertile (*n =* 38) (5.68 ± 0.31)	2nd Tertile (*n =* 38) (6.72 ± 0.28)	3rd Tertile (*n =* 38) (7.78 ± 0.53)	***p*** value^*^
**Age (years)**					**0.027**
50.0–59.9	15 (13.2)	2 (13.3)	4 (26.7)	9 (60.0)	
60.0–69.9	71 (62.3)	22 (31.0)	24 (33.8)	25 (35.2)	
≥ 70.0	28 (24.6)	14 (50.0)	10 (35.7)	4 (14.3)	
**Marital status**					0.358
Lives with partner	59 (51.8)	21 (35.6)	16 (27.1)	22 (37.3)	
Lives without partner	55 (48.2)	17 (30.9)	22 (40.0)	16 (29.1)	
**Race/color**					0.582 ^Ŧ^
White	43 (37.7)	15 (34.9)	16 (37.2)	12 (27.9)	
Black	9 (7.9)	2 (22.2)	4 (44.4)	3 (33.3)	
Yellow	6 (5.3)	4 (66.7)	1 (16.7)	1 (16.7)	
Brown	56 (49.1)	17 (30.4)	17 (30.4)	22 (39.3)	
**Education**					0.096
< 4 years	47 (41.2)	17 (36.2)	12 (25.5)	18 (38.3)	
4–8 years	32 (28.1)	8 (25.0)	17 (53.1)	7 (21.9)	
> 8 years	35 (30.7)	13 (37.1)	9 (25.8)	13 (37.1)	
**Smoking**					0.569 ^Ŧ^
Never smoked	79 (69.3)	27 (34.2)	28 (35.4)	24 (30.4)	
Make use	6 (5.3)	3 (50.0)	2 (33.3)	1 (16.7)	
Ex smoker	29 (25.4)	8 (27.6)	8 (27.6)	13 (44.8)	
**Alcohol consumption**					0.830
Never consumed	72 (63.2)	26 (36.1)	23 (31.9)	23 (31.9)	
Consume	15 (13.2)	5 (33.3)	4 (26.7)	6 (40.0)	
Consumed in the past	27 (23.7)	7 (25.9)	11 (40.7)	9 (33.3)	
**Physical Activity Level**					0.629 ^Ŧ^
Insufficiently active	53 (46.5)	19 (35.8)	19 (35.8)	15 (28.3)	
Sufficiently active	61 (53.5)	19 (31.1)	19 (31.1)	23 (37.7)	
**Menopause Time**					0.299
≤ 19 years old	59 (51.8)	16 (27.1)	20 (33.9)	23 (39.0)	
> 19 years old	55 (48.2)	22 (40.0)	18 (32.7)	15 (27.3)	
**Number of comorbidities**					1.000
1 to 3 comorbidities	85 (74.6)	28 (32.9)	29 (34.1)	28 (32.9)	
≥ 4 comorbidities	29 (25.4)	10 (34.5)	9 (31.0)	10 (34.5)	
**Polypharmacy**					0.164
1 to 4 medications	66 (57.9)	27 (40.9)	20 (30.3)	19 (28.8)	
≥ 5 medications	48 (42.1)	11 (22.9)	18 (37.5)	19 (39.6)	
**Calcium supplementation**					0.105
Make use	68 (59.6)	28 (41.2)	20 (29.4)	20 (29.4)	
Does not use	46 (40.4)	10 (21.7)	18 (39.1)	18 (39.1)	

^*^Chi-square test. ^Ŧ^ Fisher’s Exact test

When analyzing the distribution of mean age, clinical and anthropometric variables and functional capacity, according to the tertiles of ASMI, significant differences were found between the variable age with the 2nd and 3rd tertiles of ASMI (*p* = 0.004) and between body mass, BMI, CC, WC and dominant APMT with all tertiles of ASMI (*p* < 0.001) (Table 2).

**Table 2:** Distribution of clinical, anthropometric, functional capacity, and body composition variables according to the appendicular skeletal muscle mass index of postmenopausal women

Variables	ASMI (kg/m^**2**^)			
	1st Tertile (*n =* 38) (5.68 ± 0.31)	2nd tertile (*n =* 38) (6.72 ± 0.28)	3rd Tertile (*n =* 38) (7.78 ± 0.53)	***p*** value
Age (years)	67.9 ± 5.71ª	66.4 ± 5.33ª^,b^	63.6 ± 5.60^b^	**0.004**
Age at menarche (years)	13.24 ± 1.70	13.7 ± 1.90	12.9 ± 1.95	0.179
Menopause age (years)	46.5 ± 6.38	48.5 ± 5.72	45.5 ± 6.13	0.099
Time of menopause (years)	21.4 ± 8.98	17.9 ± 8.33	18.1 ± 8.91	0.162
Height (m)	1.54 ± 6.76	1.55 ± 6.43	1.54 ± 6.39	0.816
Body mass (kg)	54.5 ± 6.90ª	67.0 ± 6.86^b^	76.3 ± 9.11^c^	**< 0.001**
BMI (kg/m ^2^)	22.9 ± 2.33ª	27.7 ± 1.96^b^	32.0 ± 3.87^c^	**< 0.001**
CC (cm)	33.4 ± 2.10ª	37.2 ± 2.60^b^	39.8 ± 2.90^c^	**< 0.001**
WC (cm)	81.7 ± 8.00^a^	92.6 ± 6.35^b^	99.9 ± 10.2^c^	**< 0.001**
DAPMT (mm)	11.2 ± 3.04ª	13.6 ± 2.80^b^	15.3 ± 3.19^c^	**< 0.001**
DHGS (kg)	21.2 ± 6.07	22.0 ± 5.15	22.5 ± 5.98	0.602
Total Body Fat (%)	41.4 ± 5.12	41.6 ± 5.21	41.6 ± 6.24	0.986
TUGT (seconds)^Ŧ^	11.5 ± 2.93	11.5 ± 2.49	12.1 ± 4.88	0.861

One-way Anova test. ^Ŧ^ Kruskal-Wallis test. *ASMI* Appendicular Skeletal Muscle Mass Index, *BMI* Body mass index, *CC* Calf circumference, *WC* Waist circumference, *DAPMT* Dominant adductor pollicis muscle thickness, *DHGS* Dominant handgrip strength, *TUG* Timed Up and Go test. ^a-c^ Mean values without a common superscript letter differ.

Table 3 shows the association between the means of biochemical variables according to the tertiles of ASMI. A significant difference was identified in plasma calcium levels (*p* = 0.003) between the 1st tertile category and the 2nd and 3rd tertiles of ASMI.

**Table 3:** Distribution of biochemical variables according to the appendicular skeletal muscle mass index of postmenopausal women

Variables	ASMI (kg/m^**2**^)				
	1st Tertile (*n =* 38) (5.68 ± 0.31)	2nd tertile (*n =* 38) (6.72 ± 0.28)	3rd Tertile (*n =* 38) (7.78 ± 0.53)	***p*** value	
Glucose (mg/dL)* ^Ŧ^	99.0 ± 16.5	107.1 ± 35.7	106.5 ± 27.1	0.183	
Glycated Hb (%)** ^Ŧ^	5.9 ± 0.67	6.3 ± 1.75	6.2 ± 0.84	0.136	
PTH (pg/ml)^Ŧ^	60.6 ± 19.8	64.7 ± 25.7	63.9 ± 21.8	0.895	
Potassium (mEq/L)*	4.6 ± 0.30	4.6 ± 0.38	4.5 ± 0.37	0.247	
Vitamin D (ng/dL)^Ŧ^	31.1 ± 7.24	30.2 ± 6.97	35.2 ± 45.4	0.861	
Ionic Calcium (mg/dL)	5.0 ± 0.31^a^	4.8 ± 0.33^b^	4.8 ± 0.27^b^	**0.003**	
Phosphorus (mg/dL)^Ŧ^	4.8 ± 6.0	3.9 ± 0.53	3.6 ± 0.47	0.293	
Magnesium (mg/dL)^Ŧ^	2.0 ± 0.25	2.1 ± 0.22	2.07 ± 0.26	0.866	
Uric acid	4.1 ± 1.15	4.2 ± 1.48	3.9 ± 1.49	0.553	
Chlorine*	102.7 ± 2.45	102.7 ± 2.96	103.2 ± 2.53	0.576	
Creatinine*** ^Ŧ^	0.77 ± 0.22	0.77 ± 0.23	0.69 ± 0.30	0.532	
Alkaline phosphatase	178.1 ± 55.3	184.3 ± 51.7	191.8 ± 51.8	0.529	
CRP (mg/L)***	6.12 ± 14.6	4.77 ± 5.4	4.7 ± 6.31	0.808	
Sodium (mEq/l) *	141.3 ± 2.31	140.9 ± 2.36	141.3 ± 2.80	0.733	

One-way Anova test. ^Ŧ^ Kruskal Wallis Test. *n = 113; **n = 107; ***n = 112. *Glycated Hb* Glycated hemoglobin, *PTH* Parathyroid hormone, *CRP* C-Reactive Protein, ^a-c^ Mean values without a common superscript letter differ.

The results of multiple logistic regression revealed that BMI, CC and DAPMT were positively associated with ASMI, even after adjustments for confounding variables (Table 4). Regarding the 1st tertile of ASMI, the variables that remained associated after the final regression model were BMI (*p* = 0.002), CC (*p* = 0.035), and DAPMT (*p* = 0.028) (Table 4). For every 1 kg/m^2^ addition in BMI, the chance of having ASMI ranked in the 1st tertile was 89% less. The same was observed for the CC and DAPMT. For each addition of 1 cm in CC and 1 mm in DAPMT, there was a 75 and 53% reduction, respectively, in the chance of ASMI being in the 1st tertile.

**Table 4:** Association between the Appendicular Skeletal Muscle Mass Index after crude and adjusted logistic regression analysis in postmenopausal women

	1st Tertile of ASMI (5.68 ± 0.31) (*n =* 38)				2nd Tertile of ASMI (6.72 ± 0.28)(*n =* 38)			
	Gross OR (95% CI)	Model 1 OR (95% CI)	Model 2 OR (95% CI)	Model 3 OR (95% CI)	Gross OR (95% CI)	Model 1 OR (95% CI)	Model 2 OR (95% CI)	Model 3 OR (95% CI)
**Variables**								
**BMI**	0.23 (0.14–0.37)	0.19 (0.08–0.48)	**0.12 (0.03–0.43)**	**0.11 (0.26–0.43)**	0.61 (0.48–0.77)	0.47 (90.29–0.76)	**0.38 (0.19–0.75)**	**0.36 (0.18–0.73)**
**CC**	0.32 (0.22–0.48)	0.45 (0.22–0.91)	**0.33 (0.12–0.88)**	**0.25 (0.56–1.01)**	0.71 (0.58–0.86)	0.89 (0.69–1.14)	**0.67 (0.46–0.96)**	**0.64 (0.43–0.93)**
**WC**	0.74 (0.67–0.82)	1.05 (0.82–1.36)	1.11(0.81–1.52)	1.12(0.80–1.54)	0.90 (0.85–0.96)	1.12 (0.97–1.30)	1.18 (0.98–1.43)	1.21 (0.99–1.48)
**DAPMT**	0.62 (0.50–0.75)	0.57 (0.39–0.84)	**0.52 (0.30–0.89)**	**0.47 (0.24–0. 92)**	0.82 (0.69–0.97)	0.75 (0.57–0.99)	0.83 (0.62–1.10)	0.83 (0.63–1.10)
**Ionic calcium**	9.89 (2.05–47.71)	5.92 (0.10–351.68)	18.31 (0.72–4651.5)	15.25 (0.82–2830.0)	1.13 (0.24–5.28)	1.27 (0.13–11.90)	2.53 (0.15–42.35)	2.01 (0.10–37.26)

*OR* Odds Ratio. Model 1: adjusted for age and color/race. Model 2: adjusted for age, race/color, time since menopause, physical activity level, smoking and alcohol consumption. Model 3: adjusted for age, race/color, time since menopause, physical activity level, smoking, alcohol consumption, calcium supplementation use and polypharmacy, *ASMI* Appendicular Skeletal Muscle Mass Index, *BMI* Body mass index, *CC* Calf circumference, *WC* Waist circumference, *DAPMT* Dominant adductor pollicis muscle thickness. Reference based on 3rd tertile of ASMI.

When the 2nd tertile was analyzed in relation to the 3rd tertile, the BMI (*p* = 0.005) and CC (*p* = 0.022) remained associated after adjustments (Table 4). For every 1 kg/m^2^ and 1 cm addition, the chance of ASMI being in the 2nd tertile decreased by 64 and 36% for to BMI and CC respectively. WC and calcium levels lost their strength of association in relation to ASMI after the adjusted models.

## Discussion

Our results showed that BMI, CC, and APMT were positively associated with ASMI, which may indicate that this variable is preserved. The results obtained in the adjusted models are consistent with previous studies that assessed the relationship between these anthropometric measures and ASMI [7, 10–12, 14, 15, 40]. A possible explanation lies on the premise that these measures are predictors of muscle mass, as they have been increasingly recommended for estimating muscle in the lower and upper limbs in clinical practice [5, 7, 14, 15].

Regardless of the well-known limitation of BMI in not determining body composition, our results showed that women classified in the 3rd ASMI tertile had higher values for this indicator. This result can be explained by the fact that BMI derives from the total body mass (fat and muscle mass), so that a greater body mass may also mean greater muscle mass [12, 20]. This result can also be explained by the fact that most women evaluated in this study are physically active, with adequate life habits, shorter menopause, few comorbidities, and low use of medications, factors that positively affect preservation and maintenance of muscle and total body mass [16–19, 41].

We also observed that with higher values in BMI, the chance of the ASMI being in the lowest tertile was reduced. In recent years, the importance of considering the muscle component, in addition to the change in total body mass, has been highlighted [13, 20]. Previous studies indicate that increasing BMI without increasing muscle mass can increase the risk of fractures and mortality [12, 13]. This association between muscle mass, BMI, and mortality was identified in the study by Abramowitz et al. [12], in which higher ASMI values were independently associated with lower mortality risk, while extreme BMI values increased the risk of death when compared to eutrophic BMI values [12]. These data reinforce the fact that adequate body mass, with a predominance of muscle rather than fat mass, can be considered a protective factor for ASMI.

In addition to body mass, we found that CC and DAPMT – indicators of muscle mass – were significantly lower among women classified in the 1st tertile of ASMI when compared to the 3rd tertile. The same was observed to CC in the 2rd tertile when compared to the 3rd tertile. Kawakami et al. [11], when analyzing the adequacy of CC and the effects of obesity and age while screening for low muscle mass in 1239 Japanese aged over 40 years, observed a positive correlation between CC and ASMI measured by DXA, regardless of obesity and age. Thus, the use of CC as a simple and accurate marker in the identification and screening of low muscle mass in clinical practice can be a low-cost alternative for this diagnosis.

Likewise, Bielemann et al. [40] observed a positive relationship between APMT and ASMI. The increase in APMT is related to body size and manual work occupation, which results in higher values of muscle mass [8, 30]. It is important to emphasize that APMT suffers low interference from subcutaneous fat, being able to effectively identify a reduction in ASMI [8, 42]. Measures such as APMT and CC are simple and easy to apply, standing out for being able to predict low appendicular muscle mass [5, 7, 10, 30, 42].

Strategies for the maintenance and/or recovery of muscle mass, through the adoption of healthy lifestyle habits and dietary interventions – such as adequate protein supply and supplementation, when necessary, may delay and/or prevent undesirable outcomes in this group, such as reduced ASMI, bone mineral density (BMD) and, consequently, functional capacity and quality of life [9, 43].

Among the variables evaluated, WC and calcium levels did not remain associated with ASMI after the final adjustments. A possible explanation lies on the fact that WC is a measure used to assess the distribution of body fat, reflecting the adipose concentration of the abdominal area [29, 44], which makes it inadequate to indicate body muscle mass.

As for calcium, we observed higher serum values among women classified in the 1st tertile of ASMI. The fact that the sample came from a Climacteric and Osteoporosis clinic may have influenced this finding, since most of them used calcium supplements. Women with low muscle mass are more likely to have low BMD [2], being commonly treated with calcium supplementation. However, after final adjustments, this parameter did not remain associated with ASMI.

The reduction in ASMI is associated with functional disability and increased mortality in the general population [4, 12], a condition that worsens among postmenopausal women due to hormonal and physiological changes common in this period [20]. On the other hand, a preserved ASMI can directly influence survival, in addition to protecting against loss of functional status arising from aging or association with other comorbidities [2, 12].

This study has as a limitation the cross-sectional design, which prevents causality, in addition to having been limited to a specific outpatient audience, making it impossible to extrapolate the data to other populations. We also highlight the state of pandemic caused by the new coronavirus, SARS-CoV-2, established during the research, which compromised it in part and prevented us from reaching the sample number. Another limitation was the fact that protein intake was not considered by the current study. Nonetheless, we adjusted different combinations of variables to ensure the reliability of the results.

As a contribution, this study is one of the few that assesses the ASMI of postmenopausal women, using a reference standard for muscle mass analysis, validated questionnaires, and biochemical variables. In this sense, the use of low-cost and common practical measures for the assessment of muscle mass reserve may prevent the loss of muscle and body mass, in addition to enabling the early screening of a possible population at risk for ASMM depletion and consequent sarcopenia.

## Conclusion

ASMI was significantly associated with BMI and muscle mass reserve indicators such as CC and DAPMT. The results demonstrate the relevance of these parameters in care protocols for postmenopausal women, as well as the need to develop health strategies aimed at preserving muscle and body mass to prevent sarcopenia.

## Data Availability

The data that support the findings of this study are available from the corresponding author on reasonable request.

## Abbreviations

SMM: Skeletal muscle mass
ASMM: Appendicular SMM
ASMI: Appendicular skeletal muscle mass index
MR: Magnetic resonance
CT: Computed tomography
DXA: Dual energy x-ray absorptiometry
BIA: Bioelectrical impedance
HRT: Hormone Replacement Therapy
ELSA Brazil: Estudo longitudinal de saúde do adulto
IPAQ: International Physical Activity Questionnaire
BMI: Body mass index
CC: Calf circumference
WC: Waist circumference
DAPMT: Dominant adductor pollicis muscle thickness
ASHT: American Society of Hand Therapists
DHGS: Dominant handgrip strength
TUG: Timed Up and Go test
Glycated Hb: Glycated hemoglobin
PTH: Parathyroid hormone
CRP: C-Reactive Protein
ESR: Erythrocyte sedimentation rate
